# Radiolabeled Human Serum Albumin Nanoparticles Co-Loaded with Methotrexate and Decorated with Trastuzumab for Breast Cancer Diagnosis

**DOI:** 10.3390/jfb14090477

**Published:** 2023-09-18

**Authors:** Meliha Ekinci, Luciana Magalhães Rebelo Alencar, André Moreni Lopes, Ralph Santos-Oliveira, Derya İlem-Özdemir

**Affiliations:** 1 Faculty of Pharmacy, Department of Radiopharmacy, Ege University, Bornova, Izmir 35040, Turkey; derya.ilem@ege.edu.tr; 2 Biophysics and Nanosystems Laboratory, Department of Physics, Federal University of Maranhão, São Luis 65065-690, Brazil; luciana.alencar@ufma.br; 3 Department of Biotechnology, Engineering School of Lorena, University of São Paulo (EEL/USP), São Paulo 12612-550, Brazil; andreml@usp.br; 4 Laboratory of Nanoradiopharmacy and Synthesis of Novel Radiopharmaceuticals, Nuclear Engineering Institute, Brazilian Nuclear Energy Commission, Rio de Janeiro 21941-906, Brazil; presidenciaradiofarmacia@gmail.com; 5 Laboratory of Radiopharmacy and Nanoradiopharmaceuticals, State University of Rio de Janeiro, Rio de Janeiro 23070-200, Brazil

**Keywords:** monoclonal antibody, folate receptor, nanotechnology, diagnosis, cancer

## Abstract

Breast cancer is a leading cause of cancer-related mortality among women worldwide, with millions of new cases diagnosed yearly. Addressing the burden of breast cancer mortality requires a comprehensive approach involving early detection, accurate diagnosis, effective treatment, and equitable access to healthcare services. In this direction, nano-radiopharmaceuticals have shown potential for enhancing breast cancer diagnosis by combining the benefits of nanoparticles and radiopharmaceutical agents. These nanoscale formulations can provide improved imaging capabilities, increased targeting specificity, and enhanced sensitivity for detecting breast cancer lesions. In this study, we developed and evaluated a novel nano-radio radiopharmaceutical, technetium-99m ([^99m^Tc]Tc)-labeled trastuzumab (TRZ)-decorated methotrexate (MTX)-loaded human serum albumin (HSA) nanoparticles ([^99m^Tc]-TRZ-MTX-HSA), for the diagnosis of breast cancer. In this context, HSA and MTX-HSA nanoparticles were prepared. Conjugation of MTX-HSA nanoparticles with TRZ was performed using adsorption and covalent bonding methods. The prepared formulations were evaluated for particle size, PDI value, zeta (ζ) potential, scanning electron microscopy analysis, encapsulation efficiency, and loading capacity and cytotoxicity on MCF-7, 4T1, and MCF-10A cells. Finally, the nanoparticles were radiolabeled with [^99m^Tc]Tc using the direct radiolabeling method, and cellular uptake was performed with the nano-radiopharmaceutical. The results showed the formation of spherical nanoparticles, with a particle size of 224.1 ± 2.46 nm, a PDI value of 0.09 ± 0.07, and a ζ potential value of −16.4 ± 0.53 mV. The encapsulation efficiency of MTX was found to be 32.46 ± 1.12%, and the amount of TRZ was 80.26 ± 1.96%. The labeling with [^99m^Tc]Tc showed a high labeling efficiency (>99%). The cytotoxicity studies showed no effect, and the cellular uptake studies showed 97.54 ± 2.16% uptake in MCF-7 cells at the 120th min and were found to have a 3-fold higher uptake in cancer cells than in healthy cells. In conclusion, [^99m^Tc]Tc-TRZ-MTX-HSA nanoparticles are promising for diagnosing breast cancer and evaluating the response to treatment in breast cancer patients.

## 1. Introduction

Breast cancer is the most common cause of mortality among women, responsible for 2.3 million women diagnosed and 685,000 deaths globally [1], nearly 15% of all female fatalities [2]. In the coming years, breast cancer is expected to surpass heart disease as the leading cause of death worldwide [3]. Thus, early diagnosis and effective treatment are important factors in the fight against breast cancer [4].

Numerous studies have been performed on using nano-radiopharmaceuticals to diagnose breast cancer [5,6,7,8,9]. By incorporating radiotracers into nanoscale carriers, nano-radiopharmaceuticals can improve imaging techniques such as positron emission tomography (PET) and single-photon emission computed tomography (SPECT). These carriers, such as liposomes, nanoparticles, or quantum dots, can enhance imaging resolution, enable multimodal imaging, and improve contrast agent delivery to the tumor site [10,11,12,13,14,15,16]. Also, these nanosystems can be engineered to specifically target biomarkers or receptors overexpressed in breast cancer cells, enabling more accurate detection and localization of tumors. Antibodies, peptides, or aptamers can be conjugated to the surface of nanoparticles, allowing them to bind selectively to tumor-specific antigens and deliver the radiotracer to the cancer cells [17,18,19,20].

A suitable targeting moiety can be added to drug delivery systems to deliver drugs to malignant cells. These targeted molecules can bind to certain tumor cell receptor types and deliver anticancer drugs to these cells only. As acceptable targeting moieties in targeted drug delivery systems, several targeting agents, including antibodies, peptides, and folic acid, may be utilized [21]. Monoclonal antibodies (mAbs) have recently been researched as targeting molecules for developing delivery systems for cytotoxic drugs [22,23]. Trastuzumab (TRZ) is an anti-epidermal growth factor receptor (HER2) mAb that is used for the treatment of breast cancers [24]. In 20–30% of human breast tumors, the HER2 is overexpressed. TRZ could induce the downregulation of HER2 receptors and internalization of the HER2 receptor. This makes TRZ a promising approach for directing cytotoxic drugs to tumor cells. The TRZ-targeted drug-carrier conjugates can internalize in tumor cells in addition to the free antibody [25].

Methotrexate (MTX), a commonly used antineoplastic drug, inhibits the activity of the cytosolic enzyme dihydrofolate reductase to exert antitumor effects. While MTX has benefits for chemotherapeutic regimens, severe, dose-related adverse effects restrict its use in clinical settings [26]. Unlike free MTX, MTX-loaded nanoparticles have demonstrated a greater antitumor impact on the tumors. Using active targeting mechanisms is an additional step to keep these systems’ increased toxicity against tumor cells while decreasing it against normal cells, minimizing their side effects [27].

Human serum albumin (HSA)-based nanoparticulate systems are biodegradable, biocompatible, and non-toxic carriers of anticancer drugs. HSA and other carriers may covalently bind to therapeutic compounds [28]. Cytotoxic drugs may have fewer negative effects if they are conjugated with carriers such as HSA. As a result, it is reasonable to assume that an antibody-cytotoxic drug-carrier combination will improve the targeted delivery of anticancer drugs to tumor cells while reducing their adverse effects [29]. Is important to notice that the use of HSA has been chosen due its high cellular uptake, targeting, biocompatibility, and low immunogenicity [30,31].

Breast cancer diagnosis is critical in determining the appropriate treatment strategy and improving patient outcomes [32]. While current diagnostic methods such as mammography, ultrasound, MRI, biopsy, and molecular biomarker analysis have been invaluable, they each come with limitations [33]. In this direction, nano-radiopharmaceuticals offer a unique approach to breast cancer diagnosis by leveraging the properties of nanoparticles and radionuclides to overcome these limitations. Thus, nano-radiopharmaceuticals (i) can increase the sensitivity and specificity of the diagnosis since they can be designed to specifically target molecular markers indicative of cancer cells [34]. By attaching radiotracers to nanoparticles, these agents can home in on tumor-specific antigens or receptors, significantly improving sensitivity and specificity; (ii) can provide accurate assessment of tumor margins, aiding the precise delineation of tumor boundaries by accumulating in cancerous tissues; (iii) have real-time imaging capabilities, allowing healthcare professionals to monitor treatment responses and disease progression over time with the use of a dynamic imaging approach; (iv) may be used in personalized treatment wherein a molecular biomarker profile from the patient is chosen, like hormone receptors; (v) are minimally invasive procedures in comparison with traditional diagnostic methods, such as biopsies, and offer an alternative by providing detailed information without the need for tissue extraction; (vi) can be utilized in precise detection of metastases, with the ability to identify even small metastatic foci helping to improve staging accuracy and guide appropriate therapeutic strategies and (vii) can be used to monitor treatment efficacy, serving as valuable tools for monitoring the response to therapy by tracking the accumulation of these agents in tumor tissues, helping to make informed decisions about continuing or modifying treatment protocols [35,36,37].

The nano-radiopharmaceuticals hold the immense potential to revolutionize breast cancer diagnosis by addressing the limitations of current methods. Their ability to provide targeted, sensitive, and real-time imaging, along with the potential for personalized treatment approaches, offers a promising avenue for improving patient care. As research advances, nano-radiopharmaceuticals may play a pivotal role in enhancing breast cancer diagnosis and treatment accuracy, efficiency, and effectiveness. The aim of this study was to develop and evaluate a new nano-radiopharmaceutical for breast cancer imaging.

## 2. Materials and Methods

### 2.1. Materials

TRZ was purchased from Roche (Basel, Switzerland). MTX was obtained from Kocak Pharma (Istanbul, Turkey). All solvents and chemicals were obtained from Merck (Darmstadt, Germany). Cell culture materials and reagents were obtained from Gibco (Grand Island, NY, USA). MCF-7, 4T1, and MCF-10A cells were obtained from the ATCC (American Type Culture Collection, Manassas, VA, USA).

### 2.2. Preparation of Nanoparticles

The synthesis of HSA nanoparticles was carried out using the method developed by Ilem-Ozdemir et al. [38], with modification of the techniques developed by Langer et al. [39], Wang et al. [40], and Sebak et al. [28].

#### 2.2.1. Preparation of HSA Nanoparticles

HSA nanoparticles were prepared using the desolvation technique. First, a solution (75 mg × mL^−1^) of 150 mg HSA in 10 mM NaCl (2 mL) was prepared, and the mixture was incubated for 2 h at 100 rpm at room temperature. Then, the pH of the system was adjusted to 9.0 by adding NaOH (0.1 N NaOH). Using an injector set (1 mL) at a height of 8 cm, ethanol (70%, 8 mL) was injected dropwise into the solution at a rate of 1 mL × min^−1^, which was mixed at 550 rpm at room temperature. After the dripping process, mixing continued for 15 min. Cross-linking was performed by adding 35 µL of glutaraldehyde (8%) to the resulting nanoparticle formulations. The formulations, left to mix at 550 rpm under room conditions, were obtained after 24 h of ultracentrifugation at 20,000 rpm for 20 min at 20 °C and washed twice. The formed nanoparticles were redispersed in 2 mL phosphate buffer solution (PBS) and stored at +4 °C.

#### 2.2.2. Preparation of MTX-HSA Nanoparticles

MTX-HSA nanoparticles were also prepared using the desolvation technique. To produce MTX-HSA nanoparticles, MTX (0.5 mg) was added to a solution of 150 mg HSA in 10 mM NaCl (75 mg × mL^−1^). The same procedure for preparing HSA nanoparticles was then applied. At the end, the formed MTX-HSA nanoparticles were stored at +4 °C.

#### 2.2.3. Preparation of TRZ-MTX-HSA Nanoparticles

Two different methods were used for the preparation of TRZ-MTX-HSA nanoparticles: the adsorption method and the covalent binding method, according to the methodology employed by Kocbek and coworkers (2007) [41].

#### 2.2.4. Adsorption Method

For the adsorption method, after ultracentrifugation, MTX-HSA nanoparticles prepared as described in “Section 2.2.2” were dispersed in 1.5 mL of pH 7.4 PBS solution (1.5 mg × mL^−1^). The nanoparticle suspension (1.5 mL) was mixed with 0.5 mL of TRZ solution (0.5 mg × mL^−1^) to ensure adsorption, and the system (2 mL) was maintained at 4 °C for 24 h. At the end of the incubation period, the TRZ-adsorbed nanoparticles and free TRZ were separated by ultracentrifugation at 10,000 rpm for 15 min at 20 °C. Unbound TRZ was removed by washing the sediment twice with pH 7.4 PBS [41]. Prepared nanoparticles were redispersed in 2 mL pH 7.4 PBS, coded as TRZ-MTX-HSA-1 nanoparticles, and stored at +4 °C.

#### 2.2.5. Covalent Binding Method

For the covalent binding method, using the carbodiimide functionalization reaction, TRZ was bonded covalently to the nanoparticle’s surface [41]. For the reaction, after ultracentrifugation, MTX-HSA nanoparticles (13.5 mg) were prepared as described in “Section 2.2.2” and were dispersed in distilled water (10 mL) for 5 min at 155 W and a frequency of 50/60 Hz using an ultrasonicator device (Bandelin HD3400, Istanbul, Türkiye). Next, 12.5 mg of 1-ethyl-3-(3-dimethylaminopropyl)carbodiimide (EDC) and 38.2 mg of N-hydroxy succinimide (NHS) were mixed with distilled water (2.5 mL), and the mixture was added to the nanoparticulate system. After stirring for 4 h at room temperature, TRZ (12.9 mg), diluted in distilled water (1 mL) was added to the mixture and stirred for 18 h. At the end of the mixing period, unbound reagents were removed by ultracentrifugation at 13,000 rpm for 5 min at 20 °C [41]. Prepared nanoparticles were redispersed in 2 mL pH 7.4 PBS, coded as TRZ-MTX-HSA-2 nanoparticles, and stored at +4 °C.

### 2.3. Characterization of Nanoparticles

#### 2.3.1. Particle Size, Distribution, and ζ Potential Analysis

The synthesized nanoparticles were assessed using a Malvern Zetasizer (Nano-ZS) (Malvern Panalytical, Worcestershire, UK) with an angle of 173° for the particle size and PDI value. The ζ potential values of the samples were evaluated using a Malvern Zetasizer at 25 °C, a dielectric constant of 78.5, a conductivity of 5 mS/cm, and a field strength of 40 V/cm using a DTS 1060C ζ cuvette (Malvern Panalytical, Worcestershire, UK). Before measurement, the nanoparticles were diluted to 1:400 with distilled water (pH 7).

#### 2.3.2. Scanning Electron Microscopy (SEM) Images

The surface properties of the samples were examined by using a Philips XL 30S FEG instrument (Philips, Amsterdam, The Netherlands). For this purpose, the samples were coated with gold–palladium (4:1) on an aluminum grid, and scanning of the coated samples was carried out at ×100,000 magnification and 10 kV incremental voltage conditions.

#### 2.3.3. Encapsulation Efficiency and Loading Capacity of Nanoparticles

To ensure precise administration of a drug delivery system, two key features are the loading capacity (LC) and encapsulation efficiency (EE) of drugs onto nanoparticles. UV–Vis spectrophotometric methods were developed to calculate the encapsulated amounts of MTX and TRZ in nanoparticle formulations. UV absorption values of MTX and TRZ between 200 and 400 nm were measured to determine the wavelength of maximum absorption (λ_max_) of MTX and TRZ, respectively. Then, MTX solutions with six different concentrations (0.5, 1.0, 2.5, 5.0, 7.5, and 10 µg × mL^−1^) were prepared using the stock solutions of MTX in 0.9% sodium chloride solution (saline). TRZ solutions with six different concentrations (50, 75, 100, 125, 150, and 200 µg × mL^−1^) were prepared using stock solutions of TRZ in saline.

The EE (%) of MTX in the formed nanoparticles was calculated using Equation (1) using UV–Vis spectrophotometer (Beckman Coulter DU^®^ 730 Life Science, Brea, CA, USA) analysis carried out using the supernatant obtained after ultracentrifugation of the nanoparticles [26]:

(1) $$\%\;\mathrm{EE}\;\left(\mathrm{MTX}\right)=\left\{\frac{\left(\mathrm{Total}\;\mathrm{amount}\;\mathrm{of}\;\mathrm{MTX}-\mathrm{The}\;\mathrm{amount}\;\mathrm{of}\;\mathrm{free}\;\mathrm{MTX}\right)}{\;\mathrm{Total}\;\mathrm{amount}\;\mathrm{of}\;\mathrm{MTX}}\right\}\times 100$$

After the prepared nanoparticles were dispersed in 5 mL of a dimethyl sulfoxide (DMSO)/water (9:1, v/v) mixture, they were ultrasonicated for 30 min to separate the TRZ in the complex. The amount of TRZ (%) in the medium was analyzed with the UV–Vis spectrophotometer method using Equation (2) [42]:

(2) $$\%\;\mathrm{EE}\;\left(\mathrm{TRZ}\right)=\left\{\frac{\left(\mathrm{Total}\;\mathrm{amount}\;\mathrm{of}\;\mathrm{TRZ}-\mathrm{The}\;\mathrm{amount}\;\mathrm{of}\;\mathrm{free}\;\mathrm{TRZ}\right)}{\;\mathrm{Total}\;\mathrm{amount}\;\mathrm{of}\;\mathrm{TRZ}}\right\}\times 100$$

Also, LC of MTX and TRZ to nanoparticles was calculated according to Equation (3) [43,44]:

(3) $$\mathrm{LC}\;\left(\%\right)=\left\{\frac{\left(\mathrm{Total}\;\mathrm{amount}\;\mathrm{of}\;\mathrm{drug}-\mathrm{Drug}\;\mathrm{amount}\;\mathrm{in}\;\mathrm{the}\;\mathrm{supernatant}\right)}{\;\mathrm{Total}\;\mathrm{formulation}\;\mathrm{weight}}\right\}\times 100$$

### 2.4. Stability Studies of Nanoparticles

The stability of the nanoparticles at 5 ± 3 °C and 25 ± 5 °C under 60 ± 5% relative humidity (RH) and at 40 ± 5 °C and 75 ± 5% RH was determined and statistically evaluated for three months.

### 2.5. Radiolabeling Studies

Radiolabeling studies were performed in the presence of different amounts of reducing (stannous chloride) agents to find the optimum radiolabeling conditions. All nanoparticle formulations were labeled with [^99m^Tc]Tc. The reducing agent solutions (0.01, 0.025, 0.05, and 0.1 mg × mL^−1^ stannous chloride in distilled water) were added to the 1 mL nanoparticle solution, separately. The [^99m^Tc]-pertechnetate solution ([^99m^Tc]NaTcO_4_) was eluted from the [^99^Mo]Mo/[^99m^Tc]Tc generator. Next, 0.1 mL of [^99m^Tc]Tc (370 MBq × mL^−1^) was mixed with the nanoparticle solution for 60 s and incubated for 15 min. RTLC assessed the labeling efficiency of the nanoparticles.

#### 2.5.1. Radio Thin Layer Chromatography

Whatman 3MM (Fisher Scientific, Waltham, MA, USA) and ITLC-SG papers (Agilent, Santa Clara, CA, USA) were used as the stationary phases, while acetone and a pyridine/acetic acid/water mixture (PAW; 3:5:1.5) were used as the mobile phases [45]. The radiochemical purity (RCP) (%) was calculated using Equation (4).

(4) $$\mathrm{RP}\;\left(\%\right)=[100-(\mathrm{Free}\;[99\mathrm{mTc}]\mathrm{Tc}\;\left(\%\right)+\mathrm{Hydrolyzed}\;[99\mathrm{mTc}]\mathrm{Tc}\;\left(\%\right)]$$

#### 2.5.2. In Vitro Stability of [^99m^Tc]Tc-Labeled Nanoparticles

The stability of the labeled nanoparticles was evaluated in saline, serum (PBS/fetal bovine serum (FBS) (1:1; v/v)), and culture medium using RTLC (BioScan AR 2000, Washington, DC, USA). The radiolabeled nanoparticle formulations (0.1 mL) were incubated with 0.4 mL saline, serum, and cell medium. To assess the stability of the radiolabeling, the samples were analyzed using RTLC.

### 2.6. Cell Culture Studies

MCF-7 (ATCC, HTB-22), 4T1 (ATCC, CRL-2539), and MCF-10A (ATCC, CRL-10317) (American Type Culture Collection, Manassas, VA, USA) cells were grown in Dulbecco’s modified Eagle’s medium (DMEM) supplemented with 10% FBS and 1% L-glutamine/penicillin in a humidified atmosphere (95%) with 5% CO_2_ at 37 °C. The cell lines were cultured in 75 cm^2^ surface area flasks until the cells reached 85–95% confluence and were seeded at a density of 6 × 10^5^ cells/well in plates.

#### 2.6.1. In Vitro Cytotoxicity Studies

The cytotoxicity of the nanoparticles was evaluated using the MTT method [46]. For this method, 0.1 mL of MCF-7, 4T1, and MCF-10A cells (6 × 10^4^ cells/well) were seeded into 96-well plates. The cells were placed in an incubator. After 24 h of incubation, the plate cell media were removed, and cytotoxicity studies were initiated. First, cells were washed with PBS (0.1 mL, pH 7.4). The nanoparticles were added to the plates at 4, 8, 12, 16, and 20 µL × well^−1^ (*n* = 6). After 24 h of incubation, cells were washed once with PBS. Then, 0.1 mL of MTT solution (5 mg × mL^−1^ in PBS) was added for 4 h at 37 °C, and DMSO (0.2 mL) was added to dissolve the blue formazan crystals. After incubation for 4 h, Equation (5) was used to calculate the cell viability values (%) by comparing the measured fluorescence values at 570 nm to those of the untreated control group:

(5) $$\mathrm{Cell}\;\mathrm{viability}\;\left(\%\right)=\left(\frac{\mathrm{The}\;\mathrm{absorbance}\;\mathrm{value}\;\mathrm{read}\;\mathrm{from}\;\mathrm{the}\;\mathrm{tested}\;\mathrm{samples}}{\mathrm{The}\;\mathrm{absorbance}\;\mathrm{value}\;\mathrm{read}\;\mathrm{from}\;\mathrm{the}\;\mathrm{control}\;\mathrm{group}}\;\right)\times 100$$

#### 2.6.2. Cell Binding Studies

Cell binding studies were performed on MCF-7, 4T1, and MCF-10A cells using radiolabeled nanoparticles ([^99m^Tc]Tc-HSA, [^99m^Tc]Tc-MTX-HSA, and [^99m^Tc]Tc-TRZ-MTX-HSA-1) and reduced/hydrolyzed (R/H)-[^99m^Tc]NaTcO_4_ (control). Radioactive samples (18.5 MBq) were incubated with cells at 37 °C. The medium was collected at 30, 60, and 120 min. Trypsin-EDTA (0.5 mL) was then added to the plates for cell collection. The 6-well plates were washed with DMEM (0.5 mL) and PBS (0.5 mL) to remove the loosely bound surface [^99m^Tc]Tc radioactivity and cells, respectively. The cells were then centrifuged at 3000 rpm for 5 min. Radioactivity in cells and cell media was measured using a gamma counter (Triathler Gamma Counter, Hidex, Turku, Finland). The cell binding (%) of the radiolabeled samples was calculated using Equation (6).

(6) $$\mathrm{Cell}\;\mathrm{binding}\;\left(\%\right)=\left(\frac{\mathrm{The}\;\mathrm{radioactivity}\;\mathrm{of}\;\mathrm{cells}}{\mathrm{Total}\;\mathrm{counted}\;\mathrm{radioactivity}}\right)\times 100$$

### 2.7. Statistical Analysis

Statistical and variance analyses of all results were performed using SPSS software version 25 (V.25, SPSS, Chicago, IL, USA). The statistical significance level was *p* < 0.05 for all analyses performed.

## 3. Results and Discussion

### 3.1. Preparation, Characterization, and Stability of Nanoparticles

All the nanoparticles were successfully developed. HSA and MTX-HSA nanoparticles were prepared using the desolvation method, TRZ-MTX-HSA-1 nanoparticles were prepared using the adsorption method, and TRZ-MTX-HSA-2 nanoparticles were prepared using the covalent binding method. The characterization properties of nanoparticles are listed in Table 1.

The particle size of nanostructured delivery systems plays a significant role in their accumulation in the tumor tissue, owing to the enhanced permeability and retention (EPR) effect. Nano-drug delivery systems must escape from the RES and stay in the bloodstream longer to achieve the EPR effect. It has been suggested that nanoparticles with an average size of 200–300 nm are optimal for extending the half-life in blood circulation [47].

According to the data, HSA nanoparticles were produced with a particle size of 183.9 ± 3.08 nm and PDI of 0.05 ± 0.02. The particle size and PDI value of MTX-HSA nanoparticles prepared by adding MTX to HSA nanoparticles were 207.5 ± 2.02 nm and 0.04 ± 0.02, respectively. The particle size of TRZ-MTX-HSA nanoparticles prepared by the adsorption method was 224.1 ± 2.46 nm, and the particle size of TRZ-MTX-HSA nanoparticles prepared by the covalent bonding method was 289.0 ± 2.62 nm. The PDI value of TRZ-MTX-HSA-1 and TRZ-MTX-HSA-2 nanoparticles was 0.09 ± 0.07 and 0.29 ± 0.05, respectively. The method developed for all nanoparticle formulations was simple and repeatable.

The in vivo performance of nanoparticles can be determined by focusing on their physicochemical characteristics. A nanometer-range PDI value should be the maximum for intravenous injection in a well-designed nanoparticular system. It has been stated that nanocarriers should have 200 nm particle size to guarantee the stability of an injectable colloidal formulation [48]. The PDI represents the degree of uniformity in the particle size distribution. The PDI scale has values between 0.0 (monodisperse) and 1.0 (polydisperse) [49]. A PDI value ≤ 0.3 or below, which is usually optimal for drug delivery applications, represents a homogeneous dispersion of particles [50,51]. Therefore, our results suggest that the TRZ-MTX-HSA nanoparticles are uniform. Additionally, it was discovered that the nanoparticle particle sizes (Table 1) were suitable for circulation for a longer period and for using the EPR effect to target the tumor preferentially.

Whether colloidal systems are stable depends critically on the ζ potential, which symbolizes the electrostatic charge on the nanoparticle surface [52]. Also, the interaction of the drug delivery system with the biological system is affected by ζ potential value. A ζ potential of less than −50 mV or more than +50 mV can prevent particle aggregation compared to uncharged particles [53]. In this study, the ζ potential value of all negatively charged nanoparticle formulations was below −50 mV.

In a study, MTX-HSA nanoparticles developed by Jain et al. [54] had a particle size of 264 ± 3.5 nm, a PDI value of 0.21 ± 0.07, and a ζ potential value of −12.3 ± 2.7 mV. Similarly, in another study, biotin-functionalized MTX-HSA nanoparticles were produced with a particle size between 111 and 145 nm, PDI value between 0.10 and 0.24, and ζ potential between −12.1 and −20.45 mV [20]. In accordance with Taheri et al. [27], TRZ-MTX-HSA nanoparticles were developed with a particle size between 123.0 ± 12.0 and 346.0 ± 11.1 nm, PDI value between 0.18 ± 0.10 and 0.24 ± 0.09, and ζ potential between −31.30 ± 1.11 and −34.20 ± 2.41 mV. Based on the literature, we successfully synthesized all nanoparticle formulations.

**Figure 1:**
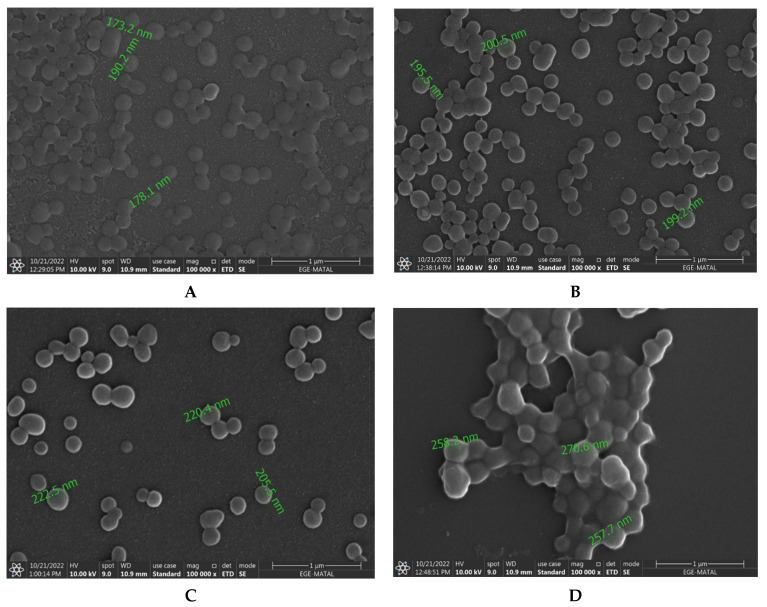
SEM images of (**A**) HSA, (**B**) MTX-HSA, (**C**) TRZ-MTX-HSA-1, and (**D**) TRZ-MTX-HSA-2 nanoparticles. A Philips XL-30S FEG brand scanning electron microscope device was used in taking this image under 100,000× magnification and 10 kV conditions.

SEM images were captured to determine the surface morphology of the nanoparticles (Figure 1).

The nanoparticles were spherical and had smooth surfaces in the obtained images (Figure 1). The resulting nanoparticles had sizes ranging from 173.2 nm to 270.6 nm, and the measurements with the Malvern ZetaSizer were in line with the results.

UV–Vis spectrophotometric methods were successfully developed to calculate the encapsulated amounts of MTX and TRZ in nanoparticle formulations. The λ_max_ of MTX was 303 nm, and the λ_max_ of TRZ was 280 nm. While the calibration curve of MTX was linear in the concentration range of 0.5–10 μg × mL^−1^ (r^2^ = 0.9998) and y = 0.0546x − 0.0191, the calibration curve of TRZ was linear in the concentration range of 50–200 μg × mL^−1^ (r^2^ = 0.9993) and y = 0.00132x − 0.00146. The EE (%) and LC (%) of MTX in the TRZ-MTX-HSA nanoparticle formulations were 32.46 ± 1.12 and 40.62 ± 2.56, respectively. The EE (%) of TRZ in TRZ-MTX-HSA nanoparticle formulations was calculated as between 75.64 ± 2.25 and 80.26 ± 1.96%, respectively. All formulations had high EE (%) and LC (%) values, and no statistical difference was observed among the nanoparticle formulations (*p* > 0.05) (Table 2). These calculated values were compatible with the literature [54].

The stability of the nanoparticles was evaluated under three storage conditions over three months, and the results are displayed in Table 3, Table 4 and Table 5. All the nanoparticles were stable and did not significantly change the characterization parameters under any of the three conditions (*p* > 0.05).

Agents such as methotrexate–human serum albumin (MTX-HSA) are commonly employed in drug delivery systems. The main goal is to enhance the therapeutic agent’s targeted delivery, stability, and efficacy while minimizing off-target effects and reducing potential toxicity. In this sense, adsorption and covalent bonding are two methods used for conjugating these agents, each with their own rationales and benefits. The adsorption involves the physical binding of molecules to the nanoparticle surface through non-covalent interactions, such as hydrogen bonding, hydrophobic interactions, and electrostatic forces. The rationales for using the adsorption method include simplicity (adsorption is a relatively straightforward method that does not require complex chemical reactions), preservation of activity (adsorption typically involves milder conditions than covalent bonding, which can help preserve the biological activity of the therapeutic agent), and flexibility (adsorption method allows for reversible interactions) [55,56,57].

Otherwise, the covalent bonding method involves forming strong chemical bonds between the therapeutic agent and the nanoparticle surface, offering stability (covalent bonds are much stronger and more stable than non-covalent interactions), specificity (covalent bonding allows for precise control over the site of attachment and the number of therapeutic agents attached to each nanoparticle), longer circulation time (covalently bound conjugates often exhibit enhanced circulation time in the bloodstream due to reduced susceptibility to degradation and clearance mechanisms), and targeting (covalent attachment can be designed to enable active targeting by incorporating targeting ligands on the nanoparticle surface) [58,59]. In the case of conjugating TRZ with MTX-HSA nanoparticles, the method chosen (adsorption or covalent bonding) will depend on factors such as the stability of TRZ under different conditions, the desired release profile of the therapeutic agent, the level of control needed over the attachment process, and the specific goals of the drug delivery system (e.g., targeted delivery, controlled release). Both methods have their merits, and researchers often select the method that best suits their intended application while considering factors such as stability, specificity, ease of preparation, and overall performance in vitro and in vivo.

The results revealed that the adsorption and covalent bonding methods used to prepare TRZ-MTX-HSA nanoparticles considerably affected the physicochemical characterization of the particles (*p* < 0.05). The most suitable method for the preparation of TRZ-MTX-HSA nanoparticles was the adsorption method, and further studies were carried out using nanoparticles prepared using this method.

### 3.2. Radiolabeling of Nanoparticles

In this study, the [^99m^Tc]Tc radionuclide, reduced to a lower oxidation valency by a reductant agent, was used for radiolabeling the nanoparticles using the direct radiolabeling approach. Using this methodology, a [^99m^Tc=O]^3+^ core was formed. The geometry of the Tc=O complex is square pyramidal, with the -yl oxygen at the apex and [^99m^Tc]Tc in the +4/+5-oxidation state. The square pyramid’s base comprises four ligands coordinating with this core [60].

**Figure 2:**
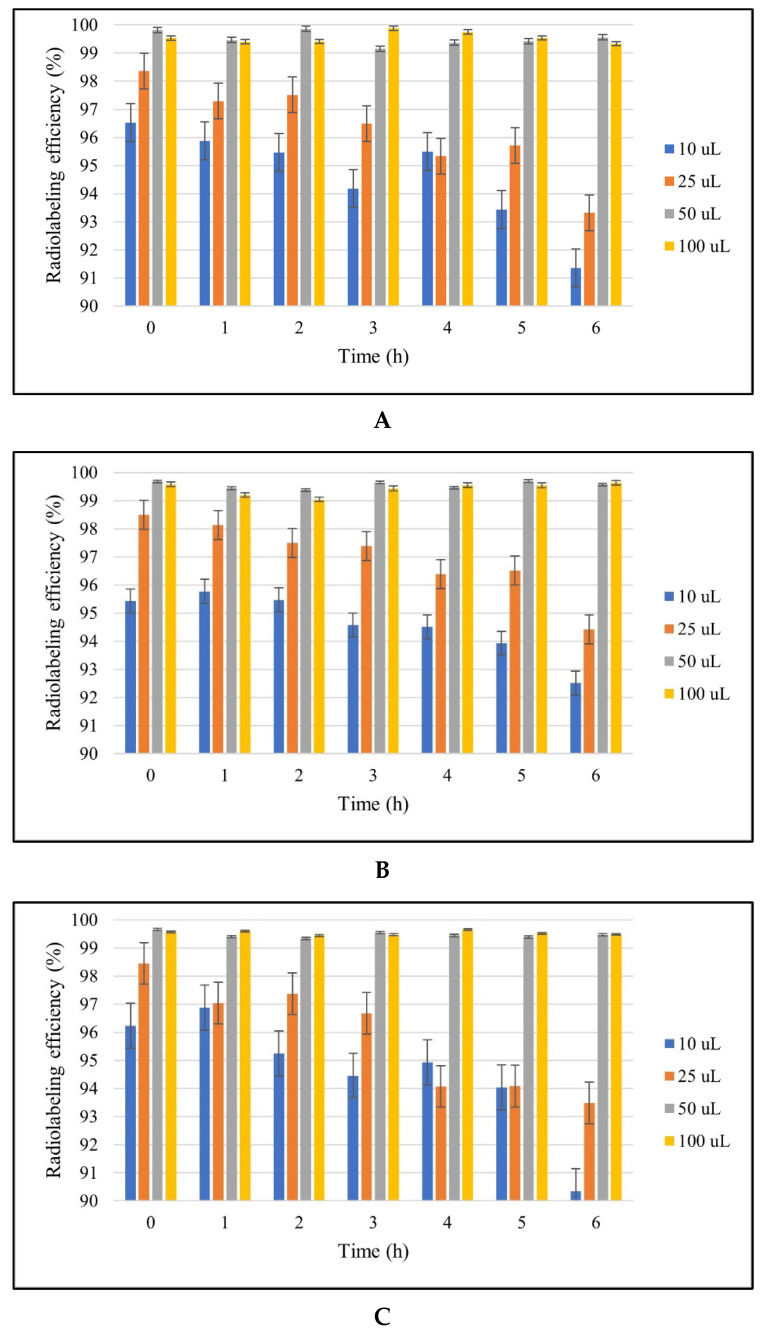
Effect of the amount of stannous chloride on the radiolabeling of nanoparticles (*n* = 3). (**A**) [^99m^Tc]Tc-HSA NPs, (**B**) [^99m^Tc]Tc-MTX-HSA NPs, (**C**) [^99m^Tc]Tc-TRZ-MTX-HAS-1 NPs.

Figure 2 illustrates the impact of different stannous chloride concentrations on the RCP of nanoparticles. As the system’s pH (pH 7.4) remained constant, stannous chloride was added in amounts ranging from 0.01 to 0.1 mg × mL^−1^ to reduce [^99m^Tc]Tc from +7 to +4/+5 valency. When 0.01 and 0.025 mg × mL^−1^ of stannous chloride were added, the RCP of the nanoparticles was above 90%. However, when 0.050 mg × mL^−1^ of stannous chloride was utilized, the RCP increased considerably to >99% (*p* < 0.05). The RCP was unaffected by a subsequent increase in stannous chloride concentration (0.1 mg × mL^−1^) (*p* > 0.05).

[^99m^Tc]Tc is the most commonly used radionuclide in radiolabeled nanostructured drug delivery systems. For radiolabeling of [^99m^Tc]Tc, the reductant agent (type and concentration) was the most important factor. Colloids form in the radiolabeling area when the amount of reducing agent is high, which lowers RCP. In contrast, free [^99m^Tc]Tc was detected in the radiolabeled area when lower doses of the reducing agent were applied. The RCP of the system was considerably affected in both situations. Most stannous salts are reductant agents in radiolabeling studies [60]. In this study, stannous chloride was used as a reductant agent for nanoparticles. The optimal concentration of stannous chloride was determined to be 0.05 mg × mL^−1^ after evaluating the effects of changing the amount of the reducing agent. In addition, 90–95% of RCP was achieved using 0.01–0.025 mg × mL^−1^ stannous chloride. The RCP of the system was unaffected by the successive addition of increasing quantities of reducing agents (*p* > 0.05). The reason for utilizing 0.05 mg × mL^−1^ of stannous chloride was based on general radiopharmacy fundamentals. Therefore, the lowest possible excipient concentration (stoichiometry) was chosen to guarantee adequate stability. The nanoparticles were incubated at 37 MBq [^99m^Tc]Tc for 6 h. The loaded amount of [^99m^Tc]Tc in nanoparticles with 0.05 mg × mL^−1^ of stannous chloride was 36.12 ± 0.02 MBq. Our results suggested that 99% of [^99m^Tc]Tc added to the nanoparticles was loaded into the nanoparticles [61,62].

Quality radiopharmaceuticals can be controlled using R-UPLC, RTLC, and/or gas chromatography [63]. A rapid and safe RTLC technique was used to test the labeling effectiveness of [^99m^Tc]Tc-nanoparticles. During [^99m^Tc]Tc labeling, three products were formed: [^99m^Tc]Tc-nanoparticles, [^99m^Tc]NaTcO_4,_ and radiocolloids. To ascertain the proportion of [^99m^Tc]NaTcO_4_ that migrated to the solvent front (R*f* = 1.0), while [^99m^Tc]Tc-nanoparticles and colloids remained at the origin (R*f* = 0.0), acetone was employed as the mobile phase, and Whatman 3MM paper was used as the stationary phase. The proportion of radiocolloids that remained at the origin (R*f* = 0.0) and migrated to the solvent front (R*f* = 1.0) was determined using a different developing solvent that contained the PAW solution (3:5:1.5). Under these conditions, the RCP of all [^99m^Tc]Tc-nanoparticles was greater than 99% (*p* < 0.05). This result was compatible with that of Jain et al. [54], who developed [^99m^Tc]Tc-MTX-has nanoparticle formulations with 98% labeling efficiency using 0.01 mg stannous chloride.

**Figure 3:**
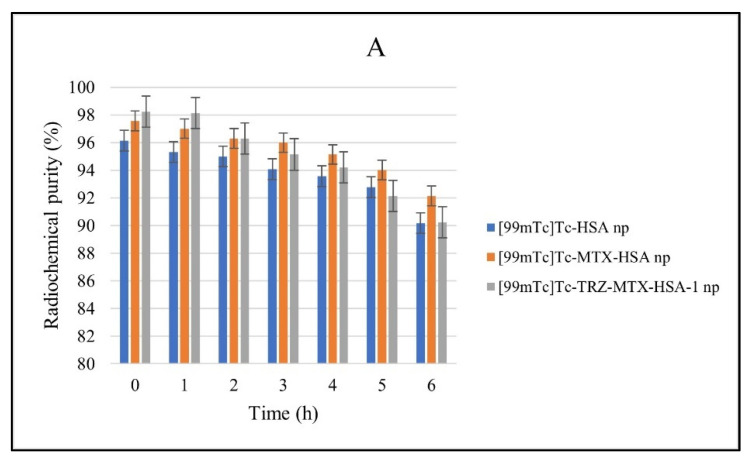

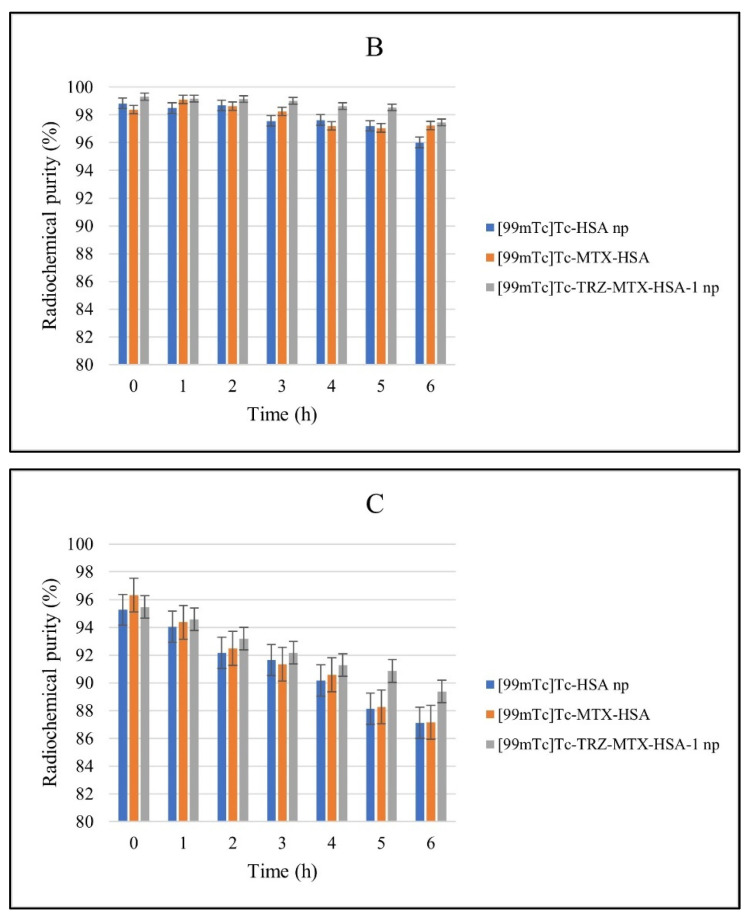
Stability of in vitro radiolabeling of nanoparticles in (**A**) saline, (**B**) cell medium, and (**C**) serum (*n* = 3).

The stability of [^99m^Tc]Tc-labeled nanoparticles was evaluated in saline, serum, and cell media (Figure 3). These parameters were selected to provide information about using [^99m^Tc]Tc-nanoparticles in an internal environment and in vitro storage [64]. As [^99m^Tc]NaTcO_4_ was eluted from the [^99^Mo]Mo/[^99m^Tc]Tc-generator using saline, [99mTc]Tc-nanoparticles must remain stable in saline. All [^99m^Tc]Tc-nanoparticles were stable in saline, with a high labeling efficiency (>90%) (Figure 3).

The stability of nano-radiopharmaceuticals plays a pivotal role in determining their potential clinical application as effective diagnostic or therapeutic agents. The stability of these nanoparticles directly affects their safety, reliability, and performance in a clinical setting. Regarding pharmacokinetics and biodistribution, stability influences how nano-radiopharmaceuticals behave in the body. For instance, unstable nanoparticles can release the radioactive payload prematurely, leading to altered pharmacokinetics and biodistribution, resulting in suboptimal targeting, reduced efficacy, and potentially increased radiation exposure to healthy tissues. Also, if nanoparticles release radioactive isotopes prematurely, it could expose healthy tissues and organs to unnecessary radiation. Moreover, unstable nanoparticles might accumulate radioactive materials in unintended sites, increasing the potential for off-target effects and toxicity. Finally, stability critically influences regulatory approval since regulatory agencies worldwide require extensive characterization of nano-radiopharmaceuticals, including stability studies, to ensure their safety, efficacy, and consistent performance [65,66,67].

When used as a tumor imaging agent and delivered in vivo, [^99m^Tc]Tc-nanoparticles must retain their stability throughout the study to accurately interpret the biodistribution and imaging results [68]. Regarding this, it was discovered that the [^99m^Tc]Tc-nanoparticles were stable in serum and had high labeling effectiveness (>87%), remaining stable for 6 h (*p* < 0.05) (Figure 3).

In addition, radiolabeled nanoparticle formulations were incubated with a cell medium for 2 h. The RCP of [^99m^Tc]Tc nanoparticles in the medium was stable, with >96% RCP (*p* < 0.05) (Figure 3). Therefore, our radiolabeled nanoparticle formulations ([^99m^Tc]Tc-HSA, [^99m^Tc]Tc-MTX-HSA, and [^99m^Tc]Tc-TRZ-MTX-HSA-1 nanoparticles) were suitable for cell incorporation studies.

### 3.3. Cell Culture Studies: Cytotoxicity and Cell Binding

**Figure 4:**
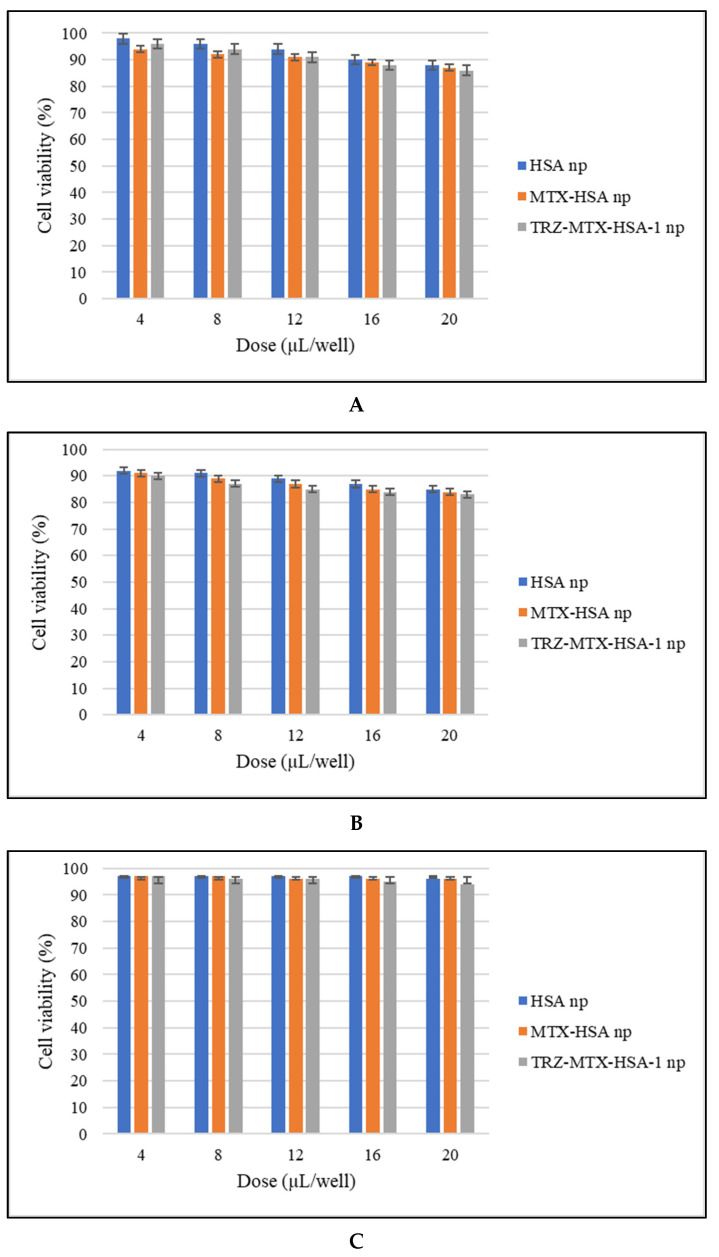
Cell viability of MCF-7 (**A**), 4T1 (**B**), and MCF-10A cells (**C**) (*n* = 6). The cells (MCF-7, 4T1, and MCF-10A) were incubated with HSA, MTX-HSA, and TRZ-MTX-HSA-1 nanoparticles in a dose-dependent manner (0–20 µL/well) for 24 h at 37 °C. Cell viability was determined using the MTT assay. DOX was used as the positive control. Experimental data are expressed as a percentage of the DMEM control. Values represent mean ± SD from three independent experiments. Statistical analyses were performed using the Mann–Whitney *U* test. *p* < 0.05 indicates statistically significant differences compared with the DMEM control.

The cytotoxicity of HSA, MTX-HSA, and TRZ-MTX-HSA-1 nanoparticle formulations in MCF-7, 4T1, and MCF-10A cells was determined by evaluating cell viability using an MTT assay. The cell viability was higher than 80% for all nanoparticle formulations (Figure 4).

The choice of cell lines for cytotoxicity studies in cancer research is critical to assessing a therapeutic agent’s potential effectiveness and selectivity against specific cancer types while considering its impact on healthy cells. In our case, the cell lines MCF-7, 4T1, and MCF-10A have been selected, and each has its rationales based on their characteristics and relevance to the study.

The MCF-7 is a well-established breast cancer cell line from the human mammary gland adenocarcinoma. It is often used in research related to breast cancer therapeutics due to the expression of estrogen and progesterone receptors, making them a model for hormone receptor-positive breast cancer. Also, these cells have low levels of HER2 expression, making them a suitable model for HER2-negative breast cancer [69,70].

The 4T1 is a murine mammary carcinoma cell line used in breast cancer research involving animal models. Its selection is based on the aggressive and metastatic behavior of these cells in mice, closely resembling the invasive characteristics of human breast cancer, making 4T1 an appropriate model for evaluating therapies targeting metastatic breast cancer [71,72,73].

Finally, the MCF-10A is an immortalized, non-transformed, human mammary epithelial cell line. It serves as a model for healthy breast tissue and is selected for control and safety assessment purposes [74,75].

In summary, the choice of MCF-7, 4T1, and MCF-10A cell lines for cytotoxicity studies is driven by the desire to evaluate the therapy’s efficacy against breast cancer, especially in hormone receptor-positive and metastatic contexts while also assessing its safety and potential impact on healthy cells. These cell lines provide valuable insights into the therapy’s potential clinical applications and limitations.

Owing to the MTX and TRZ contents, MTX-HSA and TRZ-MTX-HSA-1 nanoparticle formulations had a slightly higher cytotoxic effect than HSA nanoparticles, but this difference was not statistically significant (*p* > 0.05). This outcome indicates that the biocompatibility of protein- and HSA-based nanoparticle formulations prevents their toxic effects on healthy cells. In the literature, it was reported that TRZ-MTX-HSA nanoparticles did not show any cytotoxic effect on the human ovarian cancer cell line (SKOV-3), human breast cancer cell line (T-47D), or human adenocarcinoma cell line (HeLa), which is in agreement with our results [22]. Therefore, for future in vivo studies, TRZ-MTX-HSA-based nanoparticles can be regarded as effective and safe drug delivery vehicles because of their high biocompatibility and non-toxic characteristics.

To shed light on in vivo research, cell culture studies have recently become more important for assessing the tumor-targeting affinities of radioactive molecules or systems [76,77]. In this study, the capacity of radiolabeled nanoparticles ([^99m^Tc]Tc-HSA, [^99m^Tc]Tc-MTX-HSA, and [^99m^Tc]Tc-TRZ-MTX-HSA-1) to bind to MCF-7, 4T1, and MCF-10A cells was investigated. The cell-binding test was performed for 2 h, owing to the available half-life of [^99m^Tc]Tc. The cell binding (%) to MCF-7, 4T1, and MCF-10A cell lines of [^99m^Tc]Tc-labeled nanoparticles and R/H-[^99m^Tc]NaTcO_4_ (as a control group) are shown in Table 6.

The radiolabeled complex’s high target/non-target ratio enables the acquisition of high-quality images and prevents radiation damage to non-target tissues. Low target/non-target ratios can harm tissues in non-targeting organs and degrade target organ imaging by localization [78]. As shown in Table 6, [^99m^Tc]Tc-TRZ-MTX-HSA-1 nanoparticles had greater cell binding activity in MCF-7 cells than [^99m^Tc]Tc-HSA, [^99m^Tc]Tc-MTX-HSA nanoparticles, and R/H-[^99m^Tc]NaTcO_4_ during the experimental period. The cell binding percentage of [^99m^Tc]Tc-TRZ-MTX-HSA-1 nanoparticles in MCF-7 cells ranged from 95.21 ± 3.25% at 30 min to 97.54 ± 2.16% at 120 min. At the same time, the cell binding percentages of [^99m^Tc]Tc-HSA and [^99m^Tc]Tc-MTX-HSA nanoparticles in MCF-7 cells were found to be 60.78 ± 2.34–73.24 ± 3.49% and 81.56 ± 2.64–85.12 ± 2.46%, respectively. These findings suggest that the [^99m^Tc]Tc-TRZ-MTX-HSA-1 nanoparticle formulations increased cellular uptake compared with [^99m^Tc]Tc-HSA and [^99m^Tc]Tc-MTX-HSA nanoparticles because of the TRZ content. In addition, the cell binding (%) of [^99m^Tc]Tc-TRZ-MTX-HSA-1 nanoparticles was found to be significantly higher than that of the other formulations owing to the targeting ability of TRZ in MCF-7 and 4T1 cell lines (*p* < 0.05) and was found to have a 3-fold higher uptake in cancer cells than in healthy cells. Cell binding (%) in MCF-10A cells did not differ significantly between the formulations (*p* > 0.05).

Also, the cell binding (%) of R/H-[^99m^Tc]NaTcO_4_ ranged from 20.36 ± 2.56% at 30 min to 24.68 ± 1.56% at 120 min in MCF-7 cells. This finding proves that our labeled nanoparticle formulations behaved differently in cell medium than R/H-[^99m^Tc]NaTcO_4_ and supported the high labeling efficiency and in vitro stability (Table 6).

## 4. Conclusions

In conclusion, [^99m^Tc]Tc-TRZ-MTX-HSA-1 nanoparticle formulations have been successfully developed as a potential drug delivery system for breast cancer diagnosis. The nanoparticle formulations exhibited suitable characterization properties regarding particle size, PDI value, ζ potential, EE (%), LC (%), and SEM images. The cytotoxicity demonstrated the safety of an imaging agent. Also, the high radiolabeling yield corroborated the use of nano-radiopharmaceutical for breast cancer, which was confirmed by cell binding assays. Although preliminary, the data displayed in this study confirm the potentiality of nano-radiopharmaceuticals, especially the nano-radiopharmaceutical built for this study, as a promising new diagnosis agent. 

## Figures and Tables

**Table 1:** Characterization properties of nanoparticles (*n* = 6).

Formulations	Particle Size (nm)	Polydispersity Index	ζ Potential (mV)
HSA NPs	183.9 ± 3.08	0.05 ± 0.02	−18.0 ± 0.69
MTX-HSA NPs	207.5 ± 2.02	0.04 ± 0.02	−17.2 ± 0.41
TRZ-MTX-HSA-1 NPs	224.1 ± 2.46	0.09 ± 0.07	−16.4 ± 0.53
TRZ-MTX-HSA-2 NPs	289.0 ± 2.62	0.29 ± 0.05	−30.9 ± 1.17

**Table 2:** Encapsulation efficiency (%) and loading capacity (%) of MTX and TRZ (*n* = 3).

Formulations	EE (%) of MTX	LC (%) of MTX	EE (%) of TRZ	LC (%) of TRZ
TRZ-MTX-has-1 NPs	32.46 ± 1.12	40.62 ± 2.56	80.26 ± 1.96	85.32 ± 1.62
TRZ-MhasHSA-2 NPs	32.46 ± 1.12	40.62 ± 2.56	75.64 ± 2.25	78.36 ± 2.47

**Table 3:** Initial, 1st, and 3rd month particle sizes (nm ± SD), PDI (±SD), and ζ potential (mV ± SD) results of nanoparticles placed in a 5 ± 3 °C stability cabinet (*n* = 6).

Formulations	T_initial_	T_1month_	T_3month_
HSA NPs	183.9 ± 3.08 nm	184.6 ± 2.20 nm	185.3 ± 2.54 nm
	0.05 ± 0.02	0.05 ± 0.01	0.09 ± 0.06
	−18.0 ± 0.69 mV	−20.3 ± 0.15 mV	−19.5 ± 1.25 mV
MTX-HSA NPs	207.5 ± 2.02 nm	210.6 ± 1.44 nm	212.2 ± 2.09 nm
	0.04 ± 0.02	0.05 ± 0.04	0.05 ± 0.04
	−17.2 ± 0.41 mV	−18.6 ± 0.55 mV	−20.5 ± 1.25
TRZ-MTX-HSA-1 NPs	224.1 ± 2.46 nm	220.0 ± 1.45 nm	226.9 ± 3.21 nm
	0.09 ± 0.07	0.09 ± 0.03	0.10 ± 0.06
	−16.4 ± 0.53 mV	−20.3 ± 1.34 mV	−19.6 ± 1.69 mV
TRZ-MTX-HSA-2 NPs	289.0 ± 2.62 nm	287.4 ± 2.19 nm	295.3 ± 2.48 nm
	0.29 ± 0.05	0.29 ± 0.03	0.31 ± 0.06
	−30.9 ± 1.17 mV	−27.5 ± 1.44 mV	−32.6 ± 2.17 mV

**Table 4:** Initial, 1st, and 3rd month particle sizes (nm ± SD), PDI (±SD), and ζ potential (mV ± SD) results of nanoparticles placed in a 25 ± 5 °C stability cabinet (*n* = 6).

Formulations	T_initial_	T_1month_	T_3month_
HSA NPs	183.9 ± 3.08 nm	189.4 ± 1.52 nm	187.4 ± 2.05 nm
	0.05 ± 0.02	0.05 ± 0.01	0.07 ± 0.05
	−18.0 ± 0.69 mV	−22.6 ± 0.63 mV	−24.3 ± 2.45 mV
MTX-HSA NPs	207.5 ± 2.02 nm	212.1 ± 1.02 nm	216.3 ± 1.52 nm
	0.04 ± 0.02	0.06 ± 0.03	0.08 ± 0.06
	−17.2 ± 0.411 mV	−19.3 ± 1.56 mV	−23.5 ± 1.38 mV
TRZ-MTX-HSA-1 NPs	224.1 ± 2.46 nm	230.3 ± 2.56 nm	235.8 ± 2.15 nm
	0.09 ± 0.07	0.10 ± 0.05	0.12 ± 0.03
	−16.4 ± 0.53 mV	−23.1 ± 1.10 mV	−20.6 ± 1.02 mV
TRZ-MTX-HSA-2 NPs	289.0 ± 2.62 nm	301.6 ± 3.21 nm	308.2 ± 2.23 nm
	0.29 ± 0.05	0.28 ± 0.07	0.32 ± 0.06
	−30.9 ± 1.17 mV	−32.4 ± 2.25 mV	−37.3 ± 0.36 mV

**Table 5:** Initial, 1st, and 3rd month particle sizes (nm ± SD), PDI (±SD), and ζ potential (mV ± SD) results of nanoparticles placed in a 40 ± 5 °C stability cabinet (*n* = 6).

Formulations	T_initial_	T_1month_	T_3month_
HSA NPs	183.9 ± 3.08 nm	192.3 ± 1.35 nm	195.3 ± 2.55 nm
	0.05 ± 0.02	0.08 ± 0.03	0.09 ± 0.04
	−18.0 ± 0.69 mV	−25.6 ± 0.88 mV	−27.3 ± 1.21 mV
MTX-HSA NPs	207.5 ± 2.02 nm	218.8 ± 2.50 nm	225.6 ± 2.05 nm
	0.04 ± 0.02	0.09 ± 0.06	0.10 ± 0.05
	−17.2 ± 0.411 mV	−26.4 ± 2.56 mV	−22.2 ± 0.671 mV
TRZ-MTX-HSA-1 NPs	224.1 ± 2.46 nm	236.7 ± 3.20 nm	245.6 ± 2.03 nm
	0.09 ± 0.07	0.11 ± 0.04	0.13 ± 0.05
	−16.4 ± 0.53 mV	−20.3 ± 0.30 mV	−25.3 ± 2.30 mV
TRZ-MTX-HSA-2 NPs	289.0 ± 2.62 nm	315.3 ± 4.50 nm	322.3 ± 3.63 nm
	0.29 ± 0.05	0.34 ± 0.10	0.35 ± 0.10
	−30.9 ± 1.17 mV	−35.6 ± 2.65 mV	−38.9 ± 1.25 mV

**Table 6:** Cell binding percentage of radiolabeled formulations (*n* = 3).

Cell Line	Time (min)	[^99m^Tc]Tc-HSA NPs	[^99m^Tc]Tc-MTX-HSA NPs	[^99m^Tc]Tc-TRZ-MTX-HSA-1 NPs	R/H [^99m^Tc]Tc
MCF-7	30	60.78 ± 2.34	81.56 ± 2.64	95.21 ± 3.25	20.36 ± 2.56
MCF-7	60	72.64 ± 2.54	83.64 ± 2.18	96.38 ± 2.48	22.14 ± 2.14
MCF-7	120	73.24 ± 3.49	85.12 ± 2.46	97.54 ± 2.16	24.68 ± 1.56
4T1	30	35.23 ± 2.16	46.37 ± 2.30	50.31 ± 2.19	16.14 ± 2.40
4T1	60	38.16 ± 1.56	48.31 ± 2.59	54.67 ± 1.92	17.29 ± 1.68
4T1	120	40.46 ± 2.30	52.49 ± 2.84	60.49 ± 1.35	18.65 ± 1.37
MCF-10A	30	19.35 ± 2.64	24.61 ± 2.31	25.64 ± 1.87	8.58 ± 1.26
MCF-10A	60	20.16 ± 1.57	25.13 ± 1.52	27.61 ± 1.69	9.56 ± 1.58
MCF-10A	120	22.17 ± 2.72	27.84 ± 1.69	30.04 ± 2.06	12.25 ± 1.37

## Data Availability

All data will be available under request.
